# Treatment Patterns of Cancer-associated Thrombosis in the Netherlands: The Four Cities Study

**DOI:** 10.1055/a-2214-8101

**Published:** 2024-01-30

**Authors:** Fleur H.J. Kaptein, Noori A.M. Guman, Susan B. Lohle, Frederikus A. Klok, Albert T.A. Mairuhu, Pieter W. Kamphuisen, Nick Van Es, Menno V. Huisman

**Affiliations:** 1 Department of Thrombosis and Hemostasis, Leiden University Medical Center, Leiden, the Netherlands; 2 Department of Vascular Medicine, Amsterdam University Medical Center Location University of Amsterdam, Amsterdam, the Netherlands; 3 Department of Pulmonary Hypertension and Thrombosis, Amsterdam Cardiovascular Sciences, Amsterdam, the Netherlands; 4 Department of Internal Medicine, Tergooi Medical Center, Hilversum, the Netherlands; 5 Department of Internal Medicine, Haga Hospital, The Hague, the Netherlands

**Keywords:** venous thromboembolism, anticoagulants, neoplasms, hemorrhage, cohort studies

## Abstract

**Background** Current guidelines recommend either low-molecular weight heparin (LMWH) or direct oral anticoagulants (DOACs) as first-line treatment in cancer-associated venous thromboembolism (VTE).

**Aim** This study aimed to investigate treatment regimens for cancer-associated VTE over the past 5 years, explore predictors for initial treatment (LMWH vs. DOAC), and to assess the risks of recurrent VTE and bleeding.

**Methods** This was a Dutch, multicenter, retrospective cohort study including consecutive patients with cancer-associated VTE between 2017 and 2021. Treatment predictors were assessed with multivariable logistic regression models. Six-month cumulative incidences for recurrent VTE and major bleeding (MB) were estimated with death as competing risk.

**Results** In total, 1,215 patients were included. The majority (1,134/1,192; 95%) started VTE treatment with anticoagulation: 561 LMWH (47%), 510 DOACs (43%), 27 vitamin K antagonist (2.3%), and 36 other/unknown type (3.0%). The proportion of patients primarily treated with DOACs increased from 18% (95% confidence interval [CI] 12–25) in 2017 to 70% (95% CI 62–78) in 2021. Poor performance status (adjusted odds ratio [aOR] 0.72, 95% CI 0.53–0.99) and distant metastases (aOR 0.61, 95% CI 0.45–0.82) were associated with primary treatment with LMWH. Total 6-month cumulative incidences were 6.0% (95% CI 4.8–7.5) for recurrent VTE and 7.0% (95% CI 5.7–8.6) for MB. During follow-up, 182 patients (15%) switched from LMWH to a DOAC, and 54 patients (4.4%) vice versa, for various reasons, including patient preference, recurrent thrombosis, and/or bleeding.

**Conclusion** DOAC use in cancer-associated VTE has increased rapidly over the past years. Changes in anticoagulation regimen were frequent over time, and were often related to recurrent thrombotic and bleeding complications, illustrating the complexity and challenges of managing cancer-associated VTE.

## Introduction

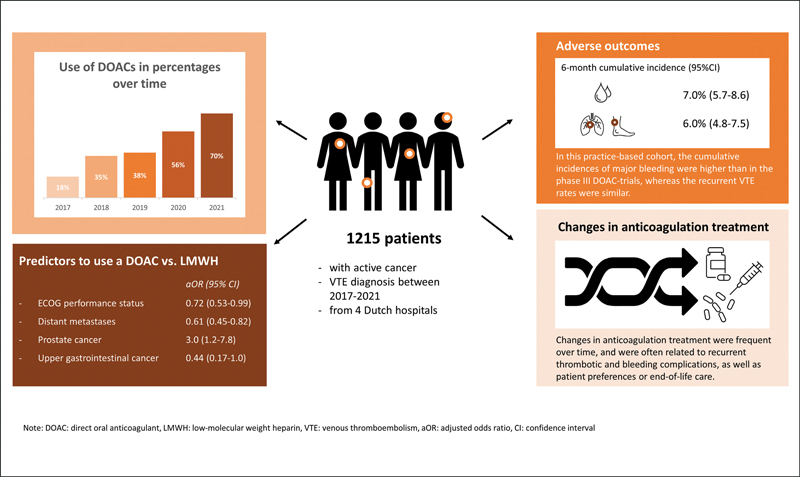



The treatment of cancer-associated venous thromboembolism (VTE) is challenging, as cancer patients more frequently develop recurrent VTE and bleeding complications during anticoagulant treatment compared to patients without cancer. 1 2 Low-molecular weight heparins (LMWH) have been standard of care for the treatment of cancer-associated VTE for years, but are burdensome because of the daily subcutaneous injections, and costly. 3 4 Multiple randomized controlled trials have demonstrated efficacy and safety of direct oral anticoagulants (DOACs) for the management of VTE in cancer patients, 5 6 7 8 and their use is now endorsed by (inter)national guidelines. 9 10 11 12 13 However, several recommendations are conditional and based on low-certainty evidence or expert opinion, for example, regarding the treatment in patients with gastrointestinal and genitourinary tumors, in which use of DOACs has been reported to be associated with a higher incidence of bleeding. 5 7 Furthermore, randomized controlled trials excluded patients with poor performance status, moderate anemia, or renal dysfunction, and the appropriate treatment in these patients is unknown.

The risk of recurrent VTE varies considerably during the course of the disease because of changing cancer treatments and frequent interventions. 14 15 Physicians are encouraged to personalize treatment decisions, 9 10 including duration and dose reductions, by considering the individual risks of bleeding and recurrence, drug–drug interactions, and patient preference, potentially resulting in heterogeneous management in clinical practice.

The aim of this study was to gain insight into the implementation of the current guidelines for cancer-associated thrombosis in daily practice in the Netherlands. We assessed the proportion of patients treated with DOACs, LMWH, or vitamin K antagonists (VKAs) over the past years, variables associated with choosing DOACs or LMWH as initial therapy, situations in which the anticoagulation therapy was adjusted, and the risks of recurrent thrombotic and bleeding complications in this vulnerable patient population.

## Methods

### Study Design, Patients, and Data Collection

In this retrospective cohort study, we included patients with active cancer who were diagnosed with acute symptomatic or incidental VTE between August 1, 2017, and May 1, 2021, in four hospitals in the Netherlands, that is, two university hospitals (Leiden University Medical Center and Amsterdam University Medical Center, Location Amsterdam Medical Center) and two nonuniversity teaching hospitals (Haga Hospital, The Hague, and Tergooi Medical Center, Hilversum).

Active cancer was defined as measurable disease and/or requiring anticancer treatment within 6 months before or after the index VTE. Both solid and hematologic malignancies were eligible. Patients with nonmalignant tumors and nonmelanoma skin cancer were excluded.

VTE comprised acute incidental or symptomatic events in any anatomical location, that is, deep vein thrombosis in extremities (including catheter-associated thrombosis) diagnosed by ultrasonography, conventional venography, or computed tomography (CT)-venography; cerebral vein thrombosis diagnosed using either CT or magnetic resonance imaging (MRI); splanchnic (portal, hepatic, splenic, and mesenteric) vein thrombosis, renal vein thrombosis or inferior vena cava thrombosis as diagnosed with ultrasonography, CT, or MRI; or incidental or symptomatic pulmonary embolism (PE; defined as at least one filling defect in the pulmonary artery tree on CT pulmonary angiography or contrast-enhanced chest CT). 16 17 18 Incidental VTE was defined as radiological confirmation of VTE on a test ordered for any other reason than suspected VTE, such as CT scans for cancer staging or treatment evaluation. Catheter-associated VTE was defined as a mural or occlusive thrombosis within the vein cannulated with the catheter or a contiguous vein.

Eligible patients were identified with use of electronic health record text mining software (CTcue B.V., Amsterdam, the Netherlands). Previous reported accuracy in the validation study of this tool was 82%, and characteristics of missed patients did not differ substantially from identified patients (i.e., missing at random). 19 In an attempt to maximize the patient identification for our study, a very sensitive search strategy with broad inclusion criteria was conducted, and all hits were verified manually ( Supplementary Table S1 , available in the online version>). Data were collected by manual review of electronic patient charts, including baseline characteristics (demographics; Eastern Cooperative Oncology Group Performance Status [ECOG] performance status; limited comorbidities; details on cancer type, stage, and treatment; and details on the index VTE and its treatment) and the outcomes of interest, using a standardized electronic case report form. Patients were followed up from the index VTE until last date of contact with a physician before the end of data collection on June 12, 2022, or until death, whichever came first.

This study was approved by the local Institutional Review Board of the four hospitals and informed consent was waived in the Leiden University Medical Center (LUMC) and Haga Hospital. As per request of the Institutional Review Board, patients from the Amsterdam University Medical Center and Tergooi MC, who were not registered as deceased, were given the opportunity to object against inclusion within 4 weeks; no additional consent was required in the other centers.

### Outcomes

We assessed the prescribing patterns for anticoagulation treatment of cancer-associated VTE, specifically anticoagulation drug class, dose, and permanent changes over time. For the latter, short interruptions because of surgery or admission were not considered as a change in anticoagulant treatment. Of note, the required LMWH lead-in with the use of edoxaban or dabigatran was disregarded. Other study outcomes were recurrent VTE, arterial thromboembolism (ATE), International Society on Thrombosis and Haemostasis-defined major bleeding (MB), clinically relevant nonmajor bleeding (CRNMB), and all-cause death. The definitions of the study outcomes are in-line with earlier studies and international guidelines and are provided in the Supplementary Material , available in the online version>. Outcomes were adjudicated independently by two of the authors (N.A.M.G. and F.H.J.K.). Discrepancies were discussed; cases in which no consensus was reached, were reviewed by a third expert (M.V.H.) for final adjudication.

### Statistical Analysis

Patient characteristics were described using means with standard deviation (SD) or median with interquartile range (IQR) for continuous variables and counts with percentages for categorical variables. Cumulative incidences of recurrent VTE, ATE, and bleeding were estimated using the cumulative incidence function in which death was considered a competing risk. 20 Patients who became lost to follow-up were included in the analyses up to the last date with available information in the patient chart. Predictors regarding treatment patterns were evaluated with binary logistic regression analysis (presented as odds ratio [OR] with 95% confidence interval [CI]). Adverse outcome predictors were evaluated with cause-specific (time-dependent) hazard models (presented as hazard ratio [HR] with 95% CI), depending on whether the time-to-event factor was relevant. For these analyses, ECOG score was divided in a good (0–1) versus a moderate-to-poor (≥2) performance status, and cancer stage in whether distant metastases were present (with hematological cancers as separate group). In the multivariable analyses, adjustment for age, sex, poor performance status, chronic pulmonary and cardiovascular comorbidity, distant metastases, and the use of chronic anticoagulation at index VTE, whereas the different cancer types and the different hospital sites as parameters were included as random effects. These results were presented as adjusted odds ratio (aOR) or adjusted hazard ratios (aHR) with 95% CIs.

All statistical analyses were performed using SPSS Statistics version 25.0 and R version 4.2.2.

## Results

### Patients

A total of 1,215 patients with cancer-associated VTE were included, with a median total follow-up time of 8.7 months (IQR 2.1–21) and a total of 1,362 patient-years of follow-up. Patient characteristics are presented in Table 1 . The mean age was 66 years (SD, 13) and 611 patients were female (50%). The most prevalent cancer types were gastrointestinal ( *n* = 386; 32%), genitourinary ( *n* = 166; 14%), and pulmonary ( *n* = 164; 13%) malignancies. The median time from (recurrent) cancer diagnosis to the index VTE was 3.3 months (IQR 0.95–11). Of the patients with a solid malignancy ( *n* = 1,088; 90%), more than half ( *n* = 601) had distant metastatic disease at the time of the VTE diagnosis.

**Table 1:** Baseline characteristics at index venous thromboembolism

Variables	*N* = 1,215
Age in years (mean, SD)	66 (13)
Female sex ( *n* , %)	611 (50.3)
Diagnosed in a university hospital ( *n* , %)	718 (59.1)
ECOG ≥ 2 ( *n* , %)	355 (30.0)
History of cardiovascular disease ( *n* , %) a	218 (17.9)
History of chronic obstructive pulmonary disease ( *n* , %) a	87 (7.2)
History of VTE ( *n* , %)	130 (10.7)
Time from (recurrent) cancer diagnosis in months (median, IQR)	3.3 (0.95–11)
Type of cancer ( *n* , %)	
Gastrointestinal	386 (31.8)
Upper gastrointestinal b	84 (6.9)
Colorectal	124 (10.2)
Pancreatic	88 (7.2)
Hepatobiliary	90 (7.4)
Lung	164 (13.5)
Gynecological	149 (12.3)
Hematological	127 (10.5)
Genitourinary (other than prostate)	94 (7.7)
Breast	90 (7.4)
Prostate	72 (5.9)
Primary brain cancer	20 (1.6)
Other	113 (9.3)
Stage of cancer ( *N* , %)	
No evidence of disease	23 (2.1)
Localized disease	225 (20.7)
Locoregional lymph node metastases	188 (17.3)
Distant metastases	457 (42.0)
Recurrent locoregional disease	51 (4.7)
Recurrent metastatic disease	144 (13.2)
Systemic anticancer therapy in previous 4 weeks ( *n* , %)	391 (32.2)
VTE type in groups ( *n* , %)	
Pulmonary embolism (with or without other VTE)	840 (69.1)
Isolated subsegmental PE	115 (9.5)
Deep vein thrombosis (with or without other VTE, except PE)	276 (22.7)
Catheter-related DVT	40 (3.3)
Isolated splanchnic vein thrombosis	80 (6.6)
Other	19 (1.6)
Incidental VTE ( *n* , %)	387 (31.9)
Use of antiplatelet agents ( *n* , %)	172 (14.2)
Use of anticoagulation ( *n* , %)	114 (9.4)
Anticoagulation started for index VTE ( *n* , %)	
None	58 (4.8)
Low-molecular weight heparin	562 (46.3)
DOAC	529 (43.5)
Apixaban	190 (15.6)
Rivaroxaban	153 (12.6)
Edoxaban	181 (14.9)
Dabigatran	5 (0.4)
Vitamin K antagonist	30 (2.5)
Unfractionated heparin	17 (1.4)
Reperfusion therapy	2 (0.2)
Antiplatelet agent	2 (0.2)
Unknown	15 (1.3)

ATE, arterial thromboembolism; DOAC, direct oral anticoagulant; DVT, deep vein thrombosis; ECOG, Eastern Cooperative Oncology Group Performance Status; IQR, interquartile range; LMWH, low-molecular weight heparin; *N* , number of patients with a solid tumor; *n* , number of total patients; PE, pulmonary embolism; SD, standard deviation; VTE, venous thromboembolism.

a Cardiovascular disease is defined as coronary artery disease, stroke, transient ischemic attack, peripheral arterial occlusion, aortic aneurysm, or chronic heart failure. Chronic obstructive pulmonary disease is defined as requiring medication.

b Upper gastrointestinal cancers includes esophagus, stomach, and upper bowel malignancies.

The most frequently diagnosed type of index VTE was PE (with or without a concurrently diagnosed different VTE type: *n* = 840; 69%), followed by deep vein thrombosis (DVT) only ( *n* = 276; 23%), and isolated splanchnic vein thrombosis ( *n* = 80; 6.6%). Almost one-third of the VTEs were diagnosed incidentally ( *n* = 387; 32%).

There were multiple differences in baseline characteristics between patients treated in the two university hospitals ( *n* = 718) and those treated in the two nonuniversity teaching hospitals ( *n* = 497; Supplementary Table S2 , available in the online version) >.

### Initial Therapeutic Treatment Regimen

In the patients who had suspected or confirmed cancer at time of the index VTE ( *n* = 1,192), LMWH was the most prescribed initial treatment agent in the total cohort ( *n* = 561, 47%), followed by a DOAC ( *n* = 510, 43%). Reperfusion therapy was started in only two patients (0.17%; both systemic thrombolysis), followed by LMWH treatment. Seventeen patients received unfractionated heparin (1.4%) as initial VTE treatment (usually hemodynamically unstable patients requiring admission to the intensive care unit). In 27 patients (2.3%), a VKA was started after an LMWH lead-in. One patient received antiplatelet therapy, and in 15 patients (1.2%) the type of anticoagulation started was unknown.

**Fig. 1:**
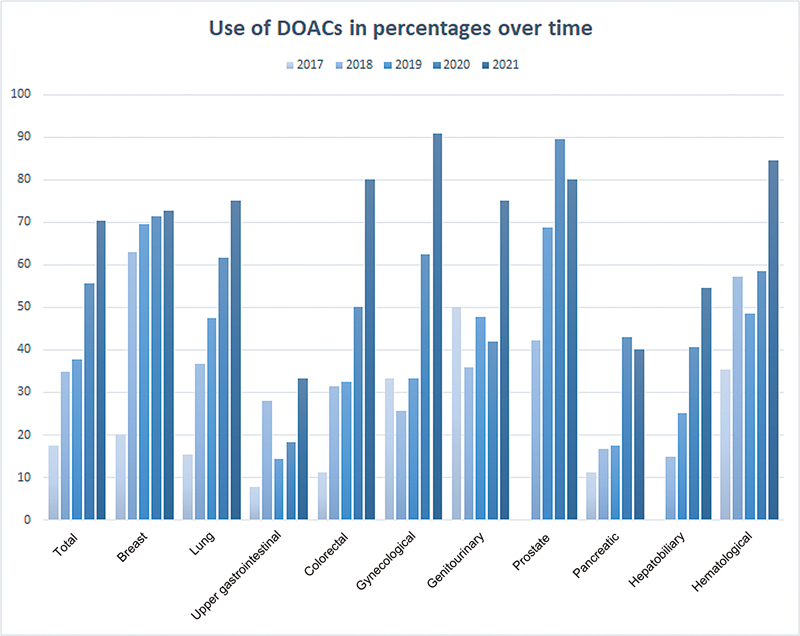
Prescription patterns per cancer site over time. DOAC, direct oral anticoagulant.

Fifty-eight patients (4.9%) did not receive any treatment for their VTE; mostly patients with isolated splanchnic VTE ( *n* = 26, of which 3 were symptomatic), incidental PE/DVT ( *n* = 7), or patients in the terminal phase ( *n* = 5). The proportion of patients treated with DOACs increased over the years, from 18% (95% CI 12–25) in 2017 to 70% (95% CI 62–78) in 2021 (aOR 1.9 per year, 95% CI 1.7–2.2). Fig. 1 shows the prescription patterns per cancer site over time.

Univariate and multivariable analyses on predictors for DOACs (vs. LMWH) as initial treatment are presented in Table 2 . DOACs were, for example, more prescribed to patients with limited disease and a better performance status. DOAC and LMWH were equally often prescribed to patients with colorectal or genitourinary cancer (excluding prostate). In upper gastrointestinal cancer, LMWH was more often prescribed (aOR 0.44, 95% CI 0.17–1.0), whereas in prostate cancer DOACs were more often used (aOR 3.0, 95% CI 1.2–7.8).

**Table 2:** Predictors for the use of direct oral anticoagulants as initial anticoagulation therapy for the index venous thromboembolism

			DOAC (vs. LMWH)	
Variable	LMWH ( *n* = 561)	DOAC ( *n* = 510)	Univariate (OR, 95% CI)	Multivariate b (aOR, 95% CI)
Age (mean, SD)	65.49 (11.65)	66.78 (13.17)	1.1 (0.99–1.2) c	1.0 (0.91–1.1) c
Female sex ( *n* , %)	281 (50.1)	266 (52.2)	1.1 (0.85–1.4)	1.0 (0.74–1.4)
ECOG ≥ 2 ( *n* , %)	175 (32.3)	120 (23.9)	0.66 (0.50–0.86)	0.72 (0.53–0.99)
Cardiovascular comorbidity a ( *n* , %)	95 (16.9)	88 (17.3)	1.0 (0.74–1.4)	1.0 (0.69–1.5)
Chronic pulmonary comorbidity a ( *n* , %)	41 (7.3)	32 (6.3)	0.85 (0.53–1.4)	0.91 (0.53–1.6)
Anticoagulation use ( *n* , %)	53 (9.5)	35 (6.9)	0.70 (0.45–1.1)	0.84 (0.51–1.4)
Antiplatelet use ( *n* , %)	76 (13.6)	78 (15.3)	1.2 (0.82–1.6)	0.95 (0.57–1.6)
eGFR < 30 mL/min/1.73 m ^2^ ( *n* , %)	10 (1.8)	7 (1.4)	0.77 (0.28–2.0)	0.73 (0.24–2.1)
Platelets < 50 × 10 ^9^ /L ( *n* , %)	8 (1.6)	5 (1.1)	1.2 (0.83–1.8)	1.4 (0.94–2.2)
Type of cancer d ( *n* , %)				
Breast	31 (5.5)	54 (10.6)	2.0 (1.3–3.2)	1.5 (0.62–3.5)
Lung	64 (11.4)	79 (15.5)	1.4 (1.0–2.0)	1.6 (0.73–3.6)
Upper gastrointestinal	61 (10.9)	17 (3.3)	0.28 (0.16–0.48)	0.44 (0.17–1.0)
Colorectal	60 (10.7)	48 (9.4)	0.87 (0.58–1.3)	0.99 (0.44–2.3)
Pancreatic	56 (10.0)	22 (4.3)	0.41 (0.24–0.67)	0.66 (0.28–1.6)
Hepatobiliary	43 (7.7)	26 (5.1)	0.65 (0.39–1.1)	1.0 (0.44–2.5)
Gynecological	73 (13.0)	64 (12.5)	0.96 (0.67–1.4)	1.3 (0.57–2.8)
Prostate	17 (3.0)	44 (8.6)	3.0 (1.7–5.5)	3.0 (1.2–7.8)
Other genitourinary	43 (7.7)	40 (7.8)	1.0 (0.65–1.6)	1.4 (0.62–3.8)
Brain	13 (2.3)	4 (0.8)	0.33 (0.093–0.95)	0.56 (0.13–2.2)
Melanoma	10 (1.8)	7 (1.4)	0.77 (0.28–2.0)	1.9 (0.52–6.8)
Sarcoma	16 (2.9)	20 (3.9)	1.4 (0.71–2.8)	2.4 (0.89–6.9)
Hematological	50 (8.9)	69 (13.5)	1.6 (1.1–2.4)	2.1 (0.94–4.7)
Distant metastases ( *N* , %)	311 (61)	225 (50.9)	0.66 (0.51–0.86)	0.61 (0.45–0.82)
Anticancer therapy d ( *n* , %)				
None	244 (43.5)	210 (41.2)	0.91 (0.71–1.2)	0.92 (0.70–1.2)
Surgery	72 (12.8)	63 (12.4)	0.96 (0.67–1.4)	1.1 (0.74–1.8)
Systemic therapy	205 (36.5)	164 (32.2)	0.82 (0.64–1.1)	0.89 (0.67–1.2)
Hormone therapy	24 (4.3)	47 (9.2)	2.3 (1.4–3.8)	1.1 (0.60–2.2)
Index VTE d ( *n* , %)				
PE (with or without concomitant other VTE type)	395 (70.4)	364 (71.4)	1.0 (0.80–1.4)	1.5 (1.1–2.0)
DVT (without concomitant PE)	127 (22.6)	124 (24.3)	1.1 (0.83–1.5)	0.67 (0.48–0.93)
Isolated splanchnic VTE	32 (5.7)	15 (2.9)	0.50 (0.26–0.92)	0.82 (0.40–1.7)
Incidental index VTE ( *n* , %)	196 (34.9)	120 (23.5)	0.57 (0.44–0.75)	0.79 (0.57–1.1)
Type of prescribing specialist d ( *n* , %)				
Internal medicine specialist	412 (73.4)	360 (70.6)	0.87 (0.66–1.1)	0.94 (0.67–1.3)
Pulmonologist	55 (9.8)	98 (19.2)	2.2 (1.5–3.1)	2.6 (1.6–4.5)
Other	94 (16.8)	51 (10.0)	0.55 (0.38–0.79)	0.58 (0.37–0.87)
University hospital ( *n* , %)	399 (71.1)	223 (43.7)	0.32 (0.24–0.41)	0.33 (0.25–0.44)

aOR, adjusted odds ratio; CI, confidence interval; DOAC, direct oral anticoagulant; DVT, deep vein thrombosis; ECOG, Eastern Cooperative Oncology Group Performance Status; eGFR, estimated glomerular filtration rate; LMWH, low-molecular weight heparin; N/A, not available; OR, odds ratio; *n* , number; PE, pulmonary embolism; SD, standard deviation; VTE, venous thromboembolism.

a Cardiovascular disease is defined as coronary artery disease, stroke, transient ischemic attack, peripheral arterial occlusion, aortic aneurysm, or chronic heart failure. Chronic obstructive pulmonary disease is defined as requiring medication.

b Adjustment for age, sex, poor performance status (ECOG ≥ 2), chronic pulmonary and cardiovascular comorbidity, distant metastases and the use of chronic anticoagulation at index VTE, whereas the different cancer types and the different hospital sites as parameters were included as random effects.

c Per 10 years increase.

d All categories are handled as binary variables (e.g., “a” vs. “non-a”).

#### Changes in Anticoagulation During Follow-up

**Fig. 2:**
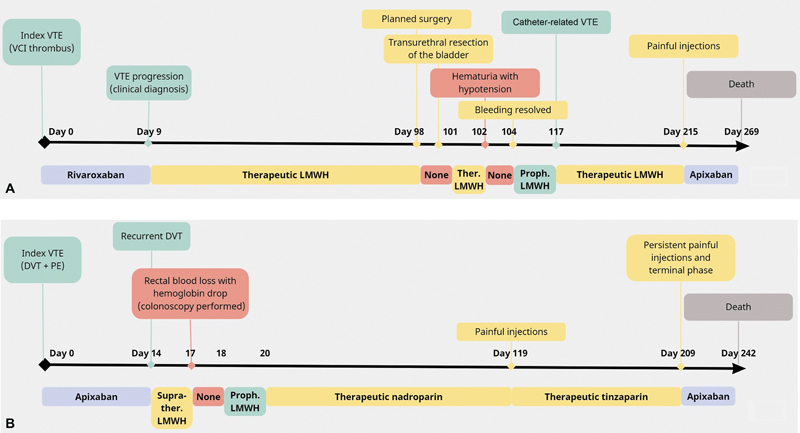
Patient's journey throughout anticoagulation treatment. ( **A** ) Patient 1 (72-year-old male with nonsmall-cell lung cancer). ( **B** ) Patient 2 (56-year-old female with colon carcinoma). DVT, deep vein thrombosis; LMWH, low-molecular weight heparin; PE, pulmonary embolism; proph., prophylactically dosed; suprather., supratherapeutically dosed (i.e., 125%); ther., therapeutically dosed; VTE, venous thromboembolism.

In 661 patients (54%), there were no changes in the anticoagulation regimen during the observation period. Table 3 shows the alterations in anticoagulation and their rationale in our cohort, with 182 patients that switched from LMWH to a DOAC (15%), and 54 patients vice versa (4.4%). In total, 84 patients (6.9%) had multiple changes during follow-up. In 243 patients (20%), anticoagulation was discontinued permanently, usually because the VTE treatment was completed when no active malignancy was present anymore (157/243, 65%). Other reasons were bleeding complications ( *n* = 41, 17%) or end-of-life care ( *n* = 28, 12%). Fig. 2 shows two examples of a timeline of a patient's journey throughout anticoagulation treatment.

**Table 3:** Changes in anticoagulation treatment during the observation period

	*N* (%)
No changes in anticoagulation	661 (54)
Switch from LMWH to DOAC	182 (15)
Initial treatment period (3–6 months) with LMWH	98
Pain/inconvenience with subcutaneous injections	68
Switch from LMWH to VKA (excluding LMWH lead-in as part of strategy)	32 (3)
Switch from DOAC to LMWH	54 (4)
Recurrent VTE	28
Inability to take oral medication	14
Bleeding	6
Reduction of anticoagulation dose	99 (8)
Half-therapeutic	83
Prophylactic	16
Unknown (participation trial)	11 (1)
Discontinuation of anticoagulation	243 (20)
Treatment completed when no active malignancy present	157
Bleeding complications	41
Terminal care	28
More than one anticoagulation change	84 (7)

DOAC, direct oral anticoagulant; LMWH, low-molecular weight heparin; *n* , number; VKA, vitamin K antagonist.

#### Adverse Outcomes

##### Recurrent Venous Thromboembolism

In total, 147 recurrent VTE events were diagnosed in 123 patients (10.1%) during follow-up, with a median time to first recurrent VTE of 4.2 months (IQR 1.3–13.5). The majority were PEs ( *n* = 69, 47%) followed by DVT ( *n* = 52, 35%). In 95 events (64%), the patient used therapeutic anticoagulation at recurrent VTE diagnosis, which was a DOAC in 43 patients (45%), LMWH in 35 (37%), VKA in 15 (16%), and unfractionated heparin (UFH) in 2 (2%).

##### Arterial Thromboembolism

Eighty-two ATEs in 65 patients (5.3%) occurred during the observation period. Most were ischemic strokes ( *n* = 53, 65%), followed by peripheral arterial occlusion ( *n* = 9, 11%) and myocardial infarction ( *n* = 8, 10%). At least 43 ATEs (52%) occurred during anticoagulation therapy; however, in 37 cases (45%) the use of anticoagulation at time of the ATE event was unknown. Only in 18 cases (22%) antiplatelet agents were started.

##### Bleeding

There were 207 bleeding events in 164 patients (13.5%) during the observation period, of which two-thirds ( *n* = 138) were MBs. The median time to first bleeding event was 1.4 months (IQR 0.33–6.7). The most prevalent location of bleeding was the gastrointestinal tract ( *n* = 88, 43%) followed by hematuria ( *n* = 22, 11%), epistaxis ( *n* = 17, 8.2%), and intracranial bleeding ( *n* = 16, 7.7%). In 163 cases, the bleeding occurred during therapeutic anticoagulation (80%; mostly LMWH [ *n* = 74; 45%] and DOAC [ *n* = 72; 44%]) and in 20 during prophylactic dose anticoagulation (10%; all LMWH [ *n* = 10] or DOAC [ *n* = 10]). Twelve patients (6%) were using an antiplatelet agent at time of bleeding, of which 7 were in combination with therapeutic anticoagulation.

##### Mortality

In total, 738 patients died during the observation period (61.3%), usually due to the malignancy ( *n* = 593, 80.4%). There were 15 (2.0%) fatal bleeds, 11 (1.5%) fatal PEs, and 6 (0.8%) fatal ATEs.

##### Cumulative Incidences and Predictors

**Fig. 3:**
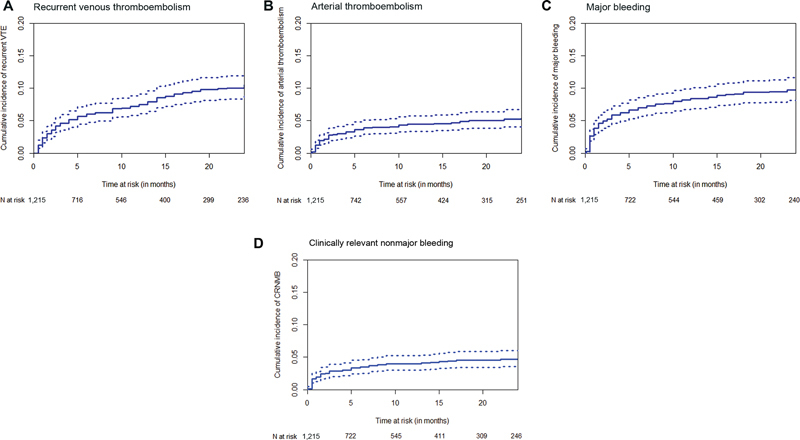
Cumulative incidences of adverse outcomes. ( **A** ) Recurrent venous thromboembolism. ( **B** ) Arterial thromboembolism. ( **C** ) Major bleeding. ( **D** ) Clinically relevant nonmajor bleeding. Cumulative incidences (solid line) with 95% confidence intervals (dashed lines) of the adverse events. ATE, arterial thromboembolism; CRNMB, clinically relevant nonmajor bleeding; *N* , number; VTE, venous thromboembolism.

Cumulative incidences of the adverse outcomes are presented in Fig. 3 . Hepatobiliary cancer was a predictor for recurrent VTE (aOR 1.9; 95% CI 1.15–3.14), whereas lung cancer was associated with ATE (aOR 2.94; 95% CI 1.57–5.51). There were no differences in risk of (major) bleeding across cancer types in multivariable analyses. Further details on predictive variables can be found in Supplementary Table S3 (available in the online version) . Multivariable time-dependent analyses showed that recurrent VTE was a predictor for subsequent bleeding in general (aHR 3.1, 95% CI 1.7–5.8). Arterial thromboembolism was a strong predictor for subsequent MB (aHR 3.5, 95% CI 1.5–8.2). Conversely, bleeding was a predictor for recurrent VTE (aHR 2.1, 95% CI 1.2–3.6). Recurrent VTE (aHR 2.8, 95% CI 2.2–3.6), ATE (aHR 3.1, 95% CI 2.2–4.2), and bleeding (i.e., both MB and CRNMB; aHR 2.3, 95% CI 1.9–2.9) were associated with mortality.

## Discussion

In the present retrospective cohort study, nearly half of the patients received a DOAC in the acute phase of cancer-associated VTE. The proportion of patients who received a DOAC increased substantially over the observation (visual summary) following accumulating evidence on the efficacy and safety of these drugs in this patient group and subsequent guideline updates.

The recurrent VTE rates in our study were comparable with those observed in the clinical trials (6.1% at 6 months vs. 5.6–7.6% in trials), 7 8 as well as in previous cohort studies (7.5% at 12 months vs. 6.5–8.7%). 21 22 23 MB rates were higher than in the randomized controlled trial (RCTs) (7.0% at 6 months vs. 3.0–5.6% in trials), probably because patients at high bleeding risk were excluded from the trials. Although the rate of bleeding varies substantially across observational studies, likely to different designs, case-mix, and definitions, our results are comparable to a study with similar outcome definitions and analyses (8.3 vs. 8.0% for MB at 12 months). 22 Arterial thromboembolism has more recently been recognized as serious complication of cancer as well, and although the rates in previous cohort studies vary, our findings are within this range (3.8% at 6 months vs. 1.1–4.7%). 23 24

In our cohort, hepatobiliary cancers were associated with a higher rate of recurrent VTE events, which has been described previously, although the pathophysiology is unclear. 25 26 A potential explanation may be that these patients more often received no anticoagulation for their index event (15.6 vs. 3.9% in nonhepatobiliary cancers), although due to low numbers no statistical analysis can be performed. Furthermore, lung cancer was a predictor for ATE in our cohort. This has also been described in other studies, 24 27 28 29 with suggested explanations as shared risk factors for cancer and ATE (e.g., smoking 24 ), genetic predisposition (e.g., ALK/ROS1 mutations 29 ), and anticancer therapy (e.g., platinum-based chemotherapy 28 ).

Comparable earlier study cohorts showed a lower DOAC use than in our study, 21 30 31 32 however all these cohorts predate 2020 (i.e., prior to the publication of the Caravaggio trial). Of note, DOACs were already used in our cohort (outside of a trial setting) before evidence from clinical trials was available. This is in line with previous studies, in line to the increased experience with these agents in the general population and the desire for oral treatment options in cancer patients. 21 32

The anticoagulation treatment of the patients in our cohort was frequently altered permanently for various reasons. Patients were often transitioned to a DOAC from an LMWH after the acute treatment phase, usually when they were deemed more stable by their clinician (e.g., more time has passed since the VTE or completion of cancer treatment) and/or to provide for patient comfort. Furthermore, changes or (temporary) discontinuation of anticoagulation due to thrombotic or bleeding complications were also common. We observed, for example, a strong correlation between the occurrence of recurrent VTE and subsequent bleeding, but also vice versa. This could be expected, as after recurrent VTE anticoagulation is usually intensified or restarted, with associated higher bleeding risk. A bleeding complication often leads to discontinuation or dose reduction of anticoagulation, resulting in a higher risk of recurrent VTE. Comparable findings were presented in a cohort study with patients with atrial fibrillation and anticoagulation, where MB was associated with a high risk of adverse (thrombotic) outcomes, part of which may be explained by anticoagulation discontinuation. 33 Furthermore, other complicating factors regarding anticoagulation use in cancer patients include cancer surgery or other invasive diagnostic or therapeutic procedures, common hospitalizations and (interactions with) systemic anticancer therapy, as well as the increased focus on quality of life in these patients, usually including minimizing their medication use and associated adverse effects. This illustrates that the treatment of cancer-associated VTE is still a major challenge, but also underlines the relevance of routinely measuring relevant outcomes beyond recurrent VTE, bleeding, and death in cancer patients with VTE: only this will allow individual patients' values and needs to be identified and incorporating in the management decision. 34

In a small but not negligible proportion of patients, the dosing of anticoagulation was reduced during extended treatment, also in the absence of general criteria for dose reduction as renal dysfunction. This strategy has been demonstrated effective in the noncancer population and therefore endorsed by guidelines, and appears to be safe in cancer patients as well, 35 although randomized controlled trials are still ongoing and conclusive evidence is yet to follow. 36 37

Strengths of our study include the multicenter design, the considerable sample size, and practice-based setting. In the absence of exclusion criteria, we believe our cohort is generalizable to the whole population of cancer-associated VTE. Although the text-mining software might not have been perfectly accurate, a very sensitive search strategy was used with manual verification, to minimize the risk of missing eligible cases. Manual review of patient charts led to rather complete collection of and detailed information on the complications. However, due to the observational design, we only had data available on the treatment within the participating hospitals. Many cancer patients are discharged from hospital care in the end-of-life stage, of which no information on outcomes was available. Our heterogeneous population is both a strength and limitation, as it provides a general overview of the management patterns over cancer-associated VTE, but the various tumor types, stages, and anticancer treatment results in small subgroups and wide CIs. As there was a lot of crossover between anticoagulation treatments, and we did not have detailed data on the timing of changes in anticoagulation available, we could not compare the different anticoagulation agents with regard to adverse outcomes.

In conclusion, our study shows that the use of DOACs in cancer-associated VTE increased rapidly over the past few years, yet changes in type of anticoagulation during treatment remain frequent. These changes often result from, but also lead to, recurrent thrombotic and bleeding complications. MBs occurred more often in our practice-based cohort than in the phase III trials, reflecting the higher risk of bleeding in an unselected population. Our results illustrate the ongoing complexity and challenges of treatment of VTE in cancer patients. Future studies on cancer-specific bleeding risk assessment models as well as on the effect of possible safer anticoagulants might contribute to better outcomes of VTE care in cancer patients.
