# Supplementary material for: Treatment Patterns of Cancer-associated Thrombosis in the Netherlands: The Four Cities Study
Source: TH Open. 2024 Jan 30;8(1):e61–71. doi: 10.1055/a-2214-8101 (PMC10827569; doi:10.1055/a-2214-8101)
Supplement: Supplementary file 1 — Supplementary Material [file 10-1055-a-2214-8101_s23080035.pdf]

Supplementary Material

Study Outcome Definitions

Recurrent venous thromboembolism was defined as one or more new filling defects on imaging test at any anatomical location compared to prior imaging. For recurrent deep vein thrombosis (DVT), the definition also included incompressibility of a new venous segment or substantial increase in vein diameter of a previous noncompressible venous segment upon compression ultrasonography, or MR direct thrombus imaging showing a high-signal intensity in a venous segment.[1]

Arterial thromboembolism included ischemic stroke, transient ischemic attack (TIA; including amaurosis fugax), myocardial infarction, and peripheral arterial embolism. Ischemic stroke was defined as an episode of neurological dysfunction caused by brain cell death attributable to ischemia, based on neuropathological, neuroimaging, and/or clinical evidence of permanent injury, in the absence of an alternative explanation, whereas a TIA was defined as a transient episode of neurological dysfunction caused by focal brain or retinal ischemia without acute infarction.[2] Myocardial infarction was defined as acute myocardial injury with clinical evidence of acute myocardial ischemia and with detection of a rise and/or fall of troponin values with at least one value above the upper limit of normal and at least one of the following: (1) symptoms of myocardial ischemia, (2) new ischemic electrocardiogram changes, (3) development of pathological Q waves, (4) imaging evidence of new loss of viable myocardium or new regional wall motion abnormality in a pattern consistent with an ischemic etiology, and (5) identification of a coronary thrombus by angiography or autopsy, as according to the Fourth Universal Definition of Myocardial Infarction.[3] Peripheral arterial embolism was defined as clinical signs of a rapid or sudden decrease in limb perfusion, with confirmation of arterial obstruction by imaging (including ultrasound, CT, MR, or conventional angiography), surgical findings, or pathology.

We followed the International Society on Thrombosis and Haemostasis definition of major bleeding (MB) and clinically relevant nonmajor bleeding (CRNMB). MB was defined as (1) fatal bleeding, (2) symptomatic bleeding in a critical area or organ, or (3) bleeding causing a fall in hemoglobin level of $\geq$1.24 mmol/L, or (4) leading to a transfusion of $\geq$2 units of blood.[4] CRNMB was defined as bleeding not fulfilling the definition of MB but meeting at least one of the following criteria: (1) requiring medical intervention by a health care professional, (2) leading to hospitalization or increased level of care, or (3) prompting a face to face (i.e., not just a telephone or electronic communication) evaluation.[5]

References

1 van Dam LF, Dronkers CEA, Gautam G, et al; Theia Study Group. Magnetic resonance imaging for diagnosis of recurrent ipsilateral deep vein thrombosis. Blood 2020;135(16):1377–1385

2 Sacco RL, Kasner SE, Broderick JP, et al; American Heart Association Stroke Council, Council on Cardiovascular Surgery and Anesthesia Council on Cardiovascular Radiology and Intervention Council on Cardiovascular and Stroke Nursing Council on Epidemiology and Prevention Council on Peripheral Vascular Disease Council on Nutrition, Physical Activity and Metabolism. An updated definition of stroke for the 21st century: a statement for healthcare professionals from the American Heart Association/American Stroke Association. Stroke 2013;44(07):2064–2089

3 Thygesen K, Alpert JS, Jaffe AS, et al; Executive Group on behalf of the Joint European Society of Cardiology (ESC)/American College of Cardiology (ACC)/American Heart Association (AHA)/World Heart Federation (WHF) Task Force for the Universal Definition of Myocardial Infarction. Fourth Universal Definition of Myocardial Infarction (2018). Circulation 2018;138(20):e618–e651

4 Schulman S, Kearon CSubcommittee on Control of Anticoagulation of the Scientific and Standardization Committee of the International Society on Thrombosis and Haemostasis. Definition of major bleeding in clinical investigations of antihemostatic medicinal products in non-surgical patients. J Thromb Haemost 2005;3(04):692–694

5 Kaatz S, Ahmad D, Spyropoulos AC, Schulman SSubcommittee on Control of Anticoagulation. Definition of clinically relevant non-major bleeding in studies of anticoagulants in atrial fibrillation and venous thromboembolic disease in non-surgical patients: communication from the SSC of the ISTH. J Thromb Haemost 2015;13(11):2119–2126

 © 2024. The Author(s).

Supplementary Table S1 Search strategy CTcue text mining software

Inclusion criteria	Sources	Content	Content for exclusion
**Age ≥ 18 years**	Demographics	Age ≥ 18 years	–
**Malignancy**			
Melanoma	ICD-10	’C43.5′, ’C43.7’, ’C43.9’	–
	DBC	’0313-842’, ’0303-350’	–
Breast	ICD-10	’C50.9’	–
	DBC	’0313-811’, ’0303-318’, ’0361-105’	–
Colorectal	ICD-10	’C18.9’, ’C20’	–
	DBC	’0313-927’ ’0313-979’, ’0303-333’, ’0303-334’, ’0303-335’, ’0361-102’	–
Brain	ICD-10	’C79.3′	–
	DBC	’0330-0202’, ’0330-0203’, ’0330-0242’	–
Gynecological	ICD-10	’C54.1’ ’C56’	–
	DBC	’0313-821’, ’0313-822’, ’0313-823’, ’0307-M11’, ’0307-M12’, ’0307-M13’, ’0307-M14’, ’0307-M15’, ’0307-M16’, ’0307-M17’, ’0307-M99’, ’0361-106’	–
Lung	ICD-10	’C34.9’, ’C78.0’	–
	DBC	’0313-621’, ’0313-622’, ’0313-623’, ’0313-624’, ’0313-629’, ’0322-1303’, ’0322-1304’, ’0322-1305’, ’0322-1306’, ’0322-1307’, ’0361-103’	–
Hepatobiliary	ICD-10	’C78.7’	–
	DBC	’0313-955’, ’0303- 331’, ’0303-367’	–
Pancreatic	ICD-10	’C25.0’, ’C25.9’	–
	DBC	’0313-964’, ’0303-332’	–
Hematological	ICD-10	’C77.9’, ’C81.9’, ’C82.9’, ’C83.3′, ’C85.9’	–
	DBC	’0313-751’, ’0313-752’, ’0313-753’, ’0313-754’, ’0313-756’, ’0313-757’, ’0313-761’, ’0313-771’, ’0313-773’	–
Esophageal or gastric	ICD-10	’C15.9’, ’C16.9’	–
	DBC	’0313-904’, ’0313-914’, ’0313-979’, ’0303-319’, ’0303-346’, ’0361-102’	–
Genitourinary	ICD-10	’C61’, ’C62.9’, ’C64’, ’C67.9’	–
	DBC	’0313-831’, ’0313-832’, ’0313-833’, ’0313-839’, ’0306-040’, ’0306-050’, ’0306-060’, ’0306-070’, ’0361-107’	–
Other	ICD-10	’C44.9’, ’C73’, ’C78.6’, ’C79.5′, ’C80.9’	–
	DBC	’0313-214’, ’0313-264’, ’0313-801, ’0313-802’, ’0313-841’, ’0313-843’, ’0313-899’, ’0303-303’, ’0303-306’ ’0303-349’, ’0303-352’, ’0303-363’, ’0305-1140’, ’0305-1150’, ’0330-0222’, ’0330-0232’, ’0361-101’, ’0361-104’, ’0361-108’, ’0361-109’	–
**Venous thromboembolism**	Radiology reports from August 1, 2017, to May 1, 2021	–	–

(*Continued*)

 © 2024. The Author(s).

Supplementary Table S1 (*Continued*)

Inclusion criteria	Sources	Content	Content for exclusion
Pulmonary embolism	CT and PET-CT	Longembolie' + 16 synonyms	Geen longembolie' + 22 synonyms
Deep vein thrombosis	Ultrasound, CT, PET-CT, and MRI	DVT' + 13 synonyms	Geen DVT' + 25 synonyms
Splanchnic and portal vein thrombosis	Ultrasound, CT, PET-CT and MRI	Porta trombose' + 6 synonyms, 'Niervene trombose' + 5 synonyms, 'V.hepatica trombose' + 4 synonyms	Geen portatrombose', 'geen v. hepatica trombose', 'geen trombose', 'geen niervene trombose', 'geen niervenetrombose'
Cerebral vein thrombosis	CT and MRI	Sinus trombose' + 6 synonyms	Geen sinustrombose' + 2 synonyms, 'geen trombose' + 2 synonyms

CT, computed tomography; DBC, Diagnosis Treatment Combination; ICD-10, 10th International Classification of Diseases; MRI, magnetic resonance imaging; PET, positron emission tomography.

 © 2024. The Author(s).

Supplementary Table S2 Baseline characteristics university versus nonuniversity teaching hospital

	Nonuniversity teaching hospital ($n = 497$)	University hospital ($n = 718$)	p-Value
Age (mean [SD])	68.77 (11.86)	64.54 (12.66)	<0.001
Female sex (%)	265 (53.3)	346 (48.2)	0.089
BMI (mean [SD])	26.49 (5.43)	26.31 (5.34)	0.569
ECOG $\geq$ 2 (%)	142 (28.6)	213 (31.0)	0.415
Cardiovascular comorbidity (%)[a]	89 (17.9)	129 (18.0)	1.000
Chronic obstructive pulmonary comorbidity (%)[a]	46 (9.3)	41 (5.7)	0.025
History of VTE (%)	45 (9.1)	85 (11.8)	0.147
Cancer type (%)			
Breast	68 (13.7)	22 (3.1)	<0.001
Lung	109 (21.9)	55 (7.7)	<0.001
Upper gastrointestinal[b]	24 (4.8)	60 (8.4)	0.023
Colorectal	61 (12.3)	63 (8.8)	0.059
Pancreatic	25 (5.0)	63 (8.8)	0.018
Hepatobiliary	13 (2.6)	77 (10.7)	<0.001
Gynecological	40 (8.0)	109 (15.2)	<0.001
Genitourinary (excluding prostate)	39 (7.8)	55 (7.7)	0.991
Prostate	41 (8.2)	31 (4.3)	0.006
Brain	3 (0.6)	17 (2.4)	0.032
Melanoma	4 (0.8)	15 (2.1)	0.124
Hematological	50 (10.1)	77 (10.7)	0.782
Sarcoma	6 (1.2)	33 (4.6)	0.002
Recurrent malignancy (%)	58 (13.0)	137 (21.3)	0.001
Distant metastases (%)[c]	272 (61.0)	329 (51.3)	0.002
Systemic anticancer therapy (%)	174 (35.0)	217 (30.2)	0.090
Platelet inhibitor use (%)	84 (16.9)	88 (12.3)	0.030
Anticoagulation use (%)	33 (6.6)	81 (11.3)	0.008
Symptomatic VTE (%)	378 (76.1)	450 (62.7)	<0.001
Type of VTE (%)			
Deep-vein thrombosis	127 (25.6)	149 (20.8)	0.058
Pulmonary embolism	347 (69.8)	491 (68.4)	0.640
Splanchnic vein thrombosis	17 (3.4)	63 (8.8)	<0.001
Other	5 (1.0)	14 (1.9)	0.285

ECOG, Eastern Cooperative Oncology Group Performance Status; n, number of total patients; SD, standard deviation; VTE, venous thromboembolism.
[a]Cardiovascular comorbidity is defined as coronary artery disease, stroke, transient ischemic attack, peripheral arterial occlusion, aortic aneurysm, or chronic heart failure. Chronic obstructive pulmonary comorbidity is defined as requiring medication.
[b]Upper gastrointestinal cancers includes esophagus, stomach, and upper bowel malignancies.
[c]Only solid tumors are included.

 © 2024. The Author(s).

Supplementary Table S3 Predictive variables of adverse outcomes

	Recurrent VTE		Bleeding (MB + CRNMB)		Major bleeding		ATE		Mortality	
	Univariate (HR, 95%CI)	Multivariate (aHR, 95%CI)	Univariate (HR, 95%CI)	Multivariate (aHR, 95%CI)	Univariate (HR, 95%CI)	Multivariate (aHR, 95%CI)	Univariate (HR, 95%CI)	Multivariate (aHR, 95%CI)	Univariate (HR, 95%CI)	Multivariate (aHR, 95%CI)
Age (per 10 years)	0.86 (0.76–0.98)	0.88 (0.76–1.02)	1.08 (0.96–1.23)	1.13 (0.98–1.29)	1.05 (0.91–1.21)	1.09 (0.94–1.28)	1.08 (0.88–1.31)	0.96 (0.76–1.20)	1.05 (0.99–1.11)	1.05 (0.98–1.12)
Female sex	0.78 (0.55–1.11)	0.77 (0.51–1.15)	0.81 (0.60–1.11)	0.94 (0.67–1.31)	0.78 (0.54–1.12)	0.86 (0.59–1.27)	1.05 (0.65–1.71)	1.52 (0.88–2.64)	0.89 (0.77–1.02)	0.94 (0.79–1.11)
BMI	1.01 (0.98–1.05)	1.02 (0.98–1.05)	1.01 (0.99–1.04)	1.02 (0.99–1.05)	1.00 (0.96–1.03)	1.00 (0.96–1.03)	0.95 (0.90–1.00)	0.96 (0.91–1.02)	0.97 (0.95–0.98)	0.98 (0.96–0.99)
University hospital	2.05 (1.37–3.07)	1.80 (1.16–2.78)	1.67 (1.19–2.33)	1.84 (1.29–2.63)	1.91 (1.27–2.87)	2.03 (1.33–3.09)	0.62 (0.38–1.00)	0.65 (0.38–1.12)	1.14 (0.98–1.32)	1.00 (0.85–1.18)
ECOG ≥ 2	1.11 (0.71–1.72)	1.03 (0.66–1.62)	1.05 (0.73–1.52)	0.91 (0.63–1.33)	1.26 (0.83–1.92)	1.11 (0.72–1.71)	1.25 (0.71–2.22)	0.99 (0.55–1.79)	2.43 (2.09–2.83)	2.27 (1.95–2.65)
Cardiovascular comorbidity[a]	1.07 (0.68–1.70)	1.05 (0.63–1.75)	1.36 (0.94–1.98)	1.15 (0.77–1.74)	1.27 (0.81–1.98)	1.04 (0.63–1.70)	2.58 (1.56–4.32)	2.51 (1.41–4.49)	1.22 (1.02–1.46)	1.17 (0.96–1.43)
Chronic pulmonary comorbidity[a]	1.07 (0.54–2.11)	0.96 (0.47–1.94)	1.08 (0.60–1.94)	0.89 (0.49–1.63)	0.86 (0.40–1.84)	0.78 (0.36–1.69)	2.58 (1.32–5.07)	1.70 (0.82–3.50)	1.05 (0.79–1.39)	0.89 (0.66–1.20)
History of VTE	0.95 (0.55–1.63)	0.92 (0.52–1.62)	0.66 (0.38–1.15)	0.51 (0.28–0.91)	0.51 (0.25–1.05)	0.37 (0.17–0.81)	0.88 (0.40–1.94)	0.89 (0.39–2.02)	0.74 (0.58–0.95)	0.66 (0.51–0.85)
**Cancer type**										
Breast	0.42 (0.18–0.95)	0.88 (0.49–1.58)	0.23 (0.09–0.62)	0.77 (0.53–1.12)	0.16 (0.04–0.63)	0.91 (0.72–1.16)	0.29 (0.07–1.21)	0.58 (0.23–1.50)	0.37 (0.26–0.54)	0.43 (0.27–0.68)
Lung	1.72 (1.08–2.73)	1.45 (0.91–2.32)	1.40 (0.92–2.14)	1.18 (0.86–1.64)	1.24 (0.74–2.08)	1.05 (0.83–1.32)	4.95 (2.99–8.17)	2.94 (1.57–5.51)	1.41 (1.16–1.72)	1.00 (0.69–1.45)
Upper GI	0.55 (0.20–1.49)	0.74 (0.41–1.34)	1.13 (0.61–2.08)	1.01 (0.70–1.44)	1.31 (0.66–2.58)	1.02 (0.81–1.29)	0.25 (0.03–1.77)	0.62 (0.23–1.72)	1.58 (1.22–2.04)	1.37 (0.92–2.05)
Colorectal	0.82 (0.44–1.52)	0.85 (0.50–1.43)	0.90 (0.54–1.51)	0.96 (0.69–1.35)	0.95 (0.52–1.73)	1.00 (0.79–1.26)	0.26 (0.06–1.08)	0.55 (0.22–1.36)	0.95 (0.75–1.20)	0.76 (0.51–1.11)
Pancreatic	1.25 (0.58–2.69)	1.09 (0.61–1.93)	1.58 (0.89–2.79)	1.17 (0.81–1.68)	1.75 (0.91–3.36)	1.06 (0.84–1.34)	1.88 (0.81–4.38)	1.85 (0.80–4.29)	2.24 (1.76–2.85)	1.89 (1.28–2.79)
Hepatobiliary	2.68 (1.62–4.44)	1.90 (1.15–3.14)	0.81 (0.41–1.58)	0.90 (0.63–1.29)	1.03 (0.50–2.11)	0.99 (0.78–1.27)	1.17 (0.47–2.92)	1.54 (0.65–3.67)	1.48 (1.15–1.90)	1.47 (0.99–2.19)
Gynecological	1.29 (0.78–2.12)	1.31 (0.79–2.16)	1.00 (0.63–1.60)	1.04 (0.74–1.46)	1.14 (0.67–1.94)	1.05 (0.83–1.32)	0.34 (0.11–1.08)	0.61 (0.25–1.49)	0.94 (0.75–1.18)	1.02 (0.69–1.51)
Genitourinary	0.81 (0.40–1.66)	0.91 (0.52–1.56)	1.64 (1.01–2.64)	1.17 (0.83–1.65)	1.38 (0.76–2.51)	1.03 (0.81–1.30)	1.70 (0.81–3.56)	1.40 (0.62–3.18)	0.94 (0.72–1.24)	0.80 (0.53–1.20)
Prostate	0.34 (0.11–1.07)	0.73 (0.40–1.36)	1.05 (0.57–1.93)	1.01 (0.70–1.46)	0.77 (0.34–1.75)	0.98 (0.77–1.24)	0.94 (0.34–2.57)	1.16 (0.47–2.90)	0.55(0.38–0.80)	0.46 (0.29–0.73)
Brain	0.70 (0.10–5.00)	0.97 (0.47–2.01)	1.44 (0.46–4.52)	1.02 (0.68–1.55)	n/a	0.97 (0.76–1.24)	n/a	0.86 (0.24–3.10)	2.33 (1.47–3.67)	3.25 (1.88–5.63)
Melanoma	1.62 (0.51–5.08)	1.06 (0.54–2.08)	1.15 (0.37–3.62)	0.95 (0.63–1.42)	1.05 (0.26–4.27)	0.98 (0.77–1.25)	1.08 (0.15–7.77)	1.05 (0.33–3.35)	1.24 (0.73–2.10)	0.76 (0.43–1.33)
Hematological	0.90 (0.52–1.54)	1.00 (0.46–2.16)	0.88 (0.55–1.43)	1.06 (0.69–1.62)	0.98 (0.57–1.68)	1.00 (0.78–1.28)	0.55 (0.22–1.38)	1.01 (0.26–3.92)	0.60 (0.46–0.78)	1.48 (0.61–3.61)
Sarcoma	0.76 (0.28–2.06)	0.83 (0.45–1.52)	0.59 (0.22–1.59)	0.88 (0.60–1.30)	0.84 (0.31–2.29)	0.98 (0.77–1.25)	n/a	0.60 (0.19–1.85)	0.68 (0.44–1.05)	0.66 (0.40–1.08)
Distant metastases	1.51 (1.03–2.21)	1.71 (1.13–2.58)	1.25 (0.90–1.74)	1.25 (0.89–1.76)	1.39 (0.94–2.06)	1.40 (0.94–2.11)	2.20 (1.29–3.77)	1.86 (1.05–3.29)	2.78 (2.36–3.27)	2.93 (2.46–3.49)
**Anticancer therapy[b]**										
None	1.90 (1.33–2.72)	2.22 (1.53–3.23)	1.28 (0.94–1.75)	1.37 (1.00–1.89)	1.29 (0.90–1.86)	1.40 (0.96–2.03)	2.07 (1.26–3.39)	2.05 (1.23–3.44)	1.62 (1.40–1.87)	1.54 (1.33–1.79)
Surgery	0.43 (0.23–0.83)	0.25 (0.12–0.52)	0.83 (0.52–1.31)	0.64 (0.38–1.07)	1.09 (0.66–1.78)	0.89 (0.51–1.56)	0.77 (0.37–1.61)	0.80 (0.34–1.92)	0.46 (0.35–0.60)	0.42 (0.31–0.56)
Systemic anticancer therapy	0.76 (0.51–1.12)	0.61 (0.40–0.95)	0.79 (0.56–1.10)	0.77 (0.54–1.10)	0.74 (0.49–1.10)	0.73 (0.48–1.12)	0.63 (0.36–1.11)	0.50 (0.27–0.92)	1.01 (0.87–1.18)	0.89 (0.76–1.05)

 © 2024. The Author(s).

Supplementary Table S3 (*Continued*)

	Recurrent VTE		Bleeding (MB + CRNMB)		Major bleeding		ATE		Mortality	
	Univariate (HR, 95%CI)	Multivariate (aHR, 95%CI)	Univariate (HR, 95%CI)	Multivariate (aHR, 95%CI)	Univariate (HR, 95%CI)	Multivariate (aHR, 95%CI)	Univariate (HR, 95%CI)	Multivariate (aHR, 95%CI)	Univariate (HR, 95%CI)	Multivariate (aHR, 95%CI)
Hormone therapy	0.37 (0.14–0.99)	0.61 (0.21–1.76)	0.98 (0.55–1.77)	1.23 (0.64–2.36)	0.64 (0.28–1.46)	0.71 (0.30–1.65)	0.57 (0.18–1.81)	0.72 (0.20–2.59)	0.42 (0.29–0.62)	0.64 (0.42–0.99)
Platelet inhibitor use	0.96 (0.57–1.63)	0.89 (0.45–1.77)	1.05 (0.68–1.64)	0.84 (0.48–1.47)	1.24 (0.76–2.03)	1.28 (0.68–2.40)	1.93 (1.09–3.44)	0.91 (0.39–2.10)	1.11 (0.90–1.36)	1.01 (0.77–1.34)
Anticoagulation use	2.07 (1.28–3.35)	2.23 (1.34–3.72)	1.12 (0.67–1.87)	0.97 (0.56–1.68)	1.18 (0.65–2.14)	1.05 (0.56–2.00)	1.91 (0.97–3.75)	1.86 (0.89–3.90)	1.14 (0.90–1.45)	1.16 (0.90–1.48)
Symptomatic VTE	0.75 (0.52–1.09)	0.91 (0.61–1.37)	0.75 (0.55–1.04)	0.85 (0.61–1.20)	0.70 (0.49–1.02)	0.80 (0.54–1.20)	1.15 (0.67–1.98)	1.31 (0.72–2.40)	0.81 (0.70–0.94)	1.06 (0.90–1.25)
**Type of index VTE**										
DVT	1.71 (1.19–2.48)	2.48 (1.66–3.71)	0.75 (0.51–1.10)	0.79 (0.56–1.11)	0.67 (0.42–1.07)	0.75 (0.47–1.22)	1.21 (0.71–2.07)	1.51 (0.85–2.68)	0.80 (0.67–0.95)	0.96 (0.80–1.16)
PE	0.46 (0.32–0.66)	0.39 (0.27–0.57)	0.96 (0.69–1.33)	0.89 (0.60–1.33)	0.92 (0.63–1.34)	0.80 (0.54–1.18)	1.05 (0.62–1.76)	0.84 (0.48–1.45)	1.10 (0.94–1.28)	1.01 (0.86–1.18)
Splanchnic vein thrombosis	1.83 (1.03–3.26)	1.46 (0.76–2.79)	1.96 (1.20–3.20)	2.13 (1.24–3.60)	2.50 (1.48–4.24)	2.64 (1.51–4.61)	0.23 (0.03–1.64)	0.25 (0.03–1.85)	1.22 (0.93–1.61)	0.95 (0.70–1.28)
Other	3.38 (1.38–8.29)	2.81 (1.00–7.88)	1.75 (0.65–4.72)	2.39 (0.87–6.59)	1.25 (0.31–5.04)	1.51 (0.37–6.21)	1.19 (0.16–8.55)	1.82 (0.24–13.68)	1.49 (0.88–2.52)	1.58 (0.90–2.77)
**Time-dependent analyses**										
Recurrent VTE	n/a	n/a	3.38 (1.84–6.18)	3.14 (1.69–5.82)	2.62 (1.25–5.47)	2.35 (1.11–4.96)	1.23 (0.38–4.01)	1.19 (0.36–3.90)	3.07 (2.45–3.86)	2.81 (2.21–3.56)
ATE	2.80 (1.22–6.40)	2.56 (1.07–6.17)	2.57 (1.13–5.84)	2.23 (0.95–5.23)	3.38 (1.48–7.75)	3.45 (1.45–8.24)	n/a	n/a	3.61 (2.68–4.86)	3.05 (2.21–4.19)
Total bleeding	2.20 (1.29–3.73)	2.06 (1.19–3.58)	n/a	n/a	n/a	n/a	1.79 (0.81–3.96)	1.16 (0.58–2.81)	2.45 (2.01–2.99)	2.32 (1.88–2.87)
Major bleeding	2.37 (1.24–4.53)	2.19 (1.12–4.28)	n/a	n/a	n/a	n/a	2.16 (0.86–5.43)	1.39 (0.49–3.96)	2.99 (2.38–3.76)	2.67 (2.10–3.39)
CRNMB	1.97 (0.91–4.23)	1.87 (0.85–4.11)	n/a	n/a	n/a	n/a	1.06 (0.26–4.38)	0.78 (0.19–3.30)	1.64 (1.19–2.26)	1.68 (1.21–2.33)

aHR, adjusted hazard ratio; ATE, arterial thromboembolism; BMI, body mass index; CI, confidence interval; CRNMB, clinically relevant nonmajor bleeding; DVT, deep vein thrombosis; ECOG, Eastern Cooperative Oncology Group Performance Status; GI, gastrointestinal; MB, major bleeding; n/a, cannot be estimated; PE, pulmonary embolism; VTE, venous thromboembolism.

[a]Cardiovascular comorbidity: defined as coronary artery disease, stroke, transient ischemic attack, peripheral arterial occlusion, aortic aneurysm, or chronic heart failure. Chronic pulmonary comorbidity: defined as obstructive pulmonary disease requiring medication.

[b]Anticancer treatment in previous 4 weeks.

 © 2024. The Author(s).
